# DCE-MRI quantitative analysis and MRI-based radiomics for predicting the early efficacy of microwave ablation in lung cancers

**DOI:** 10.1186/s40644-025-00851-7

**Published:** 2025-03-10

**Authors:** Chen Yang, Fandong Zhu, Jing Yang, Min Wang, Shijun Zhang, Zhenhua Zhao

**Affiliations:** 1 Department of Radiology, Shaoxing People’s Hosipital, Shaoxing, China; 2 Department of Pathology, School of Medicine, Zhongda Hospital, Southeast University, Nanjing, China

**Keywords:** DCE-MRI, Lung cancer, Microwave ablation, Radiomics, Quantitative analysis

## Abstract

**Objectives:**

To evaluate the feasibility and value of dynamic contrast-enhanced MRI (DCE-MRI) quantitative analysis and MRI-based radiomics in predicting the efficacy of microwave ablation (MWA) in lung cancers (LCs).

**Methods:**

Forty-three patients with LCs who underwent DCE-MRI within 24 h of receiving MWA were enrolled in the study and divided into two groups according to the modified response evaluation criteria in solid tumors (m-RECIST) criteria: the effective treatment (complete response + partial response + stable disease, *n* = 28) and the ineffective treatment (progressive disease, *n* = 15). DCE-MRI datasets were processed by Omni. Kinetics software, using the extended tofts model (ETM) and exchange model (ECM) to yield pharmacokinetic parameters and their histogram features. Changes in quantitative perfusion parameters were compared between the two groups. Scientific research platform (https://medresearch.shukun.net/) was used for radiomics analysis. A total of 1874 radiomic features were extracted for each tumor by manually segmentation of T1WI and Contrast-enhanced of T1WI (Ce-T1WI) fat inhibition sequence. The performances of radiomics models were evaluated by the receiver operating characteristic curve. Based on radiomics features, survival curves were generated by Kaplan-Meier survival analysis to evaluate patient outcomes. *P* < 0.05 was set for the significance threshold.

**Results:**

The V_p_ value of ECM was significantly higher in the ineffective group compared to the effective group (*p* = 0.027). Additionally, the skewness, and kurtosis of V_p_ (*p* = 0.020 and 0.013, respectively) derived from ETM and F_p_ (*p* = 0.027 and 0.030, respectively) from ECM as well as the quantiles were higher in the ineffective group than in the effective group. Significant statistical differences were observed in the energy and entropy of V_e_ (*p* = 0.044 and 0.025, respectively) and V_p_ (*p* = 0.025 and 0.026, respectively) between the two groups. In the process of model construction, seven features from T1WI, five features from Ce-T1WI, and ten features from combined sequences were ultimately selected. The area under the curve (AUC) values for the T1WI model, Ce-T1WI model, and combined model were 0.910, 0.890, 0.965 in the training group, and 0.850, 0.700, 0.850 in the test group, respectively.

**Conclusions:**

DCE-MRI quantitative analysis and MRI-based radiomics may be helpful in assessing the early response to MWA in patients with LCs.

**Supplementary Information:**

The online version contains supplementary material available at 10.1186/s40644-025-00851-7.

## Introduction

Lung cancer (LC) is known to have the highest mortality rate with the poor prognosis among all tumors [1]. For patients with lung metastasis or non-small cell lung cancer (NSCLC) who are not suitable for surgery, microwave ablation (MWA) is a viable treatment option due to its minimally invasive nature, good prognosis, and fewer complications [2, 3]. The ground glass opacity (GGO) of the ablation zone refers to the hazy area of increased density on imaging, often seen on CT scans or MRI, that surrounds the ablated region after MWA or other forms of thermal ablation therapy for tumors. The GGO represents a mixture of viable and non-viable tumor cells, inflammatory exudates, and coagulative necrosis within the ablation zone. Studies have shown that the presence and extent of GGO around the ablation zone could be used as an early indicator of treatment efficacy [4]. It suggested that the target tissue had been adequately covered during the ablation procedure. While CT scans are commonly used to visualize GGO [5], distinguishing GGO from bleeding or other complications in the immediate post-operative period can be challenging but is crucial for accurate assessment of treatment outcomes and timely intervention if necessary [6].

MRI, with its higher soft tissue contrast, might offer better discrimination between these entities. Lin et al. [7] posited that exudates could be observed on MRI, while isodensity was noted on CT. MRI may be performed in addition to CT when the ablation zone is not obvious. In some cases, patchy areas of high signal are seen within and/or around the ablation area on pre-contrast T1 images, most likely corresponding to ablation-related haemorrhage, which may compensate for CT deficiencies. Roman et al. [8] discovered that enhanced MRI performed within 24 h after MWA could effectively delineate the ablation zone and the primary tumor boundary. The ablation zone has intact tissue necrosis in the central region and viable tissue in the peripheral rim [9]. The peripheral margin shows hyperdensity on unenhanced CT, strong hyperdensity on T2 sequence, and a ring of enhancement on Contrast-enhanced T1 (Ce-T1) sequence, and boundary of tumor shows better on Ce-T1 and T2 than on CT. In addition, Chen et al. [10] found that MRI evaluation of RFA in the treatment of small lung malignancies (< 3 cm) had an accurate and reliable curative effect. Consequently, MRI demonstrates certain advantages in visualizing post-ablation lung lesions. Dynamic contrast-enhanced MRI (DCE-MRI) not only elucidates the morphological characteristics of the lesion but also reflects the hemodynamic changes subsequent to treatment [11]. DCE-MRI pharmacokinetic (PK) parameters quantitatively reflect tissue perfusion and blood vascular density, and have been extensively employed to predict early treatment response in various tumors, predominantly in brain and breast tumors [12], with recent applications in liver tumors [13], lung tumors [14], and osteoid osteomas [15]. Pulmonary diseases are characterized by dual blood supply from the pulmonary artery and bronchial artery [16]. Following ablation, coagulative necrosis occurs, and the ensuing hemodynamic alterations may serve as predictors of early therapeutic efficacy.

Moreover, emerging techniques in radiomics, which involve the high-throughput extraction and analysis of quantitative features from medical images, may potentially provide more precise methods for characterizing the GGO and predicting the treatment outcome [17]. Previous investigations have utilized CT-based radiomics model to predict the early postoperative efficacy [18] and local progression [19] of MWA in lung malignancies. Nevertheless, there are challenges in identifying GGO from adjacent bleeding on CT images when manually mapping the region of interest (ROI). The MRI-based radiomics model has been successfully implemented to predict the efficacy of MWA in rectal cancer [20], liver metastasis of rectal cancer [21], and small liver cancer [22]. It has previously been reported that the radiomics model of multi-parameter MRI possessed the potential to differentiate the histological grade of NSCLC [23]. Therefore, we propose that MRI-based radiomics may hold the potential to predict the early efficacy of MWA in lung malignancies.

This study deliberated on the feasibility and value of DCE-MRI quantitative analysis and MRI-based radiomics in predicting the efficacy of MWA in lung malignancies, to optimize treatment strategies and improve patient outcomes.

## Methods

### Participants

The research was approved by the ethics committee of our institution (No. IEC-K-AF-060-1.0) and written informed consent for DCE-MRI, MWA and subsequent examinations was obtained from our participants.

**Fig. 1:**
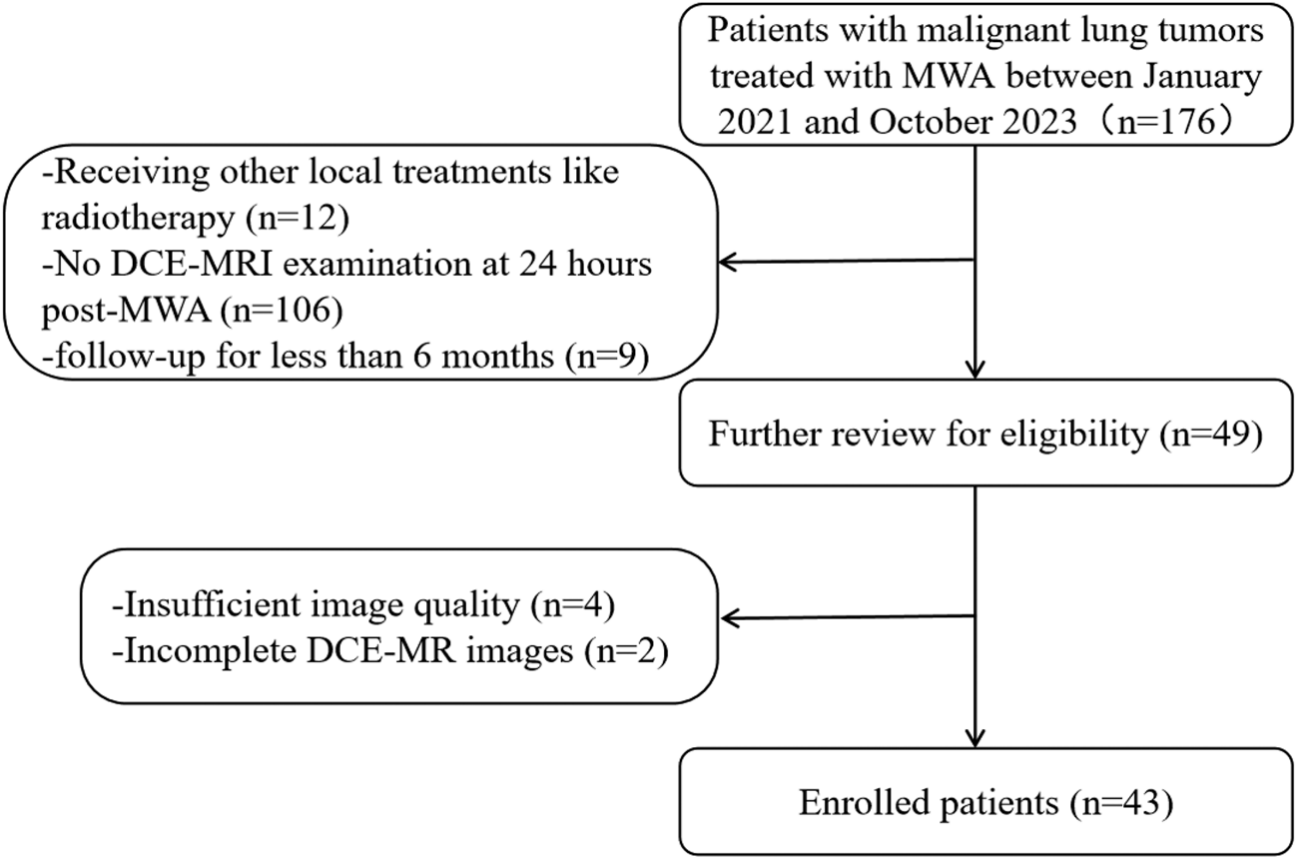
Flow chart of the patient selection process

**Fig. 2:**
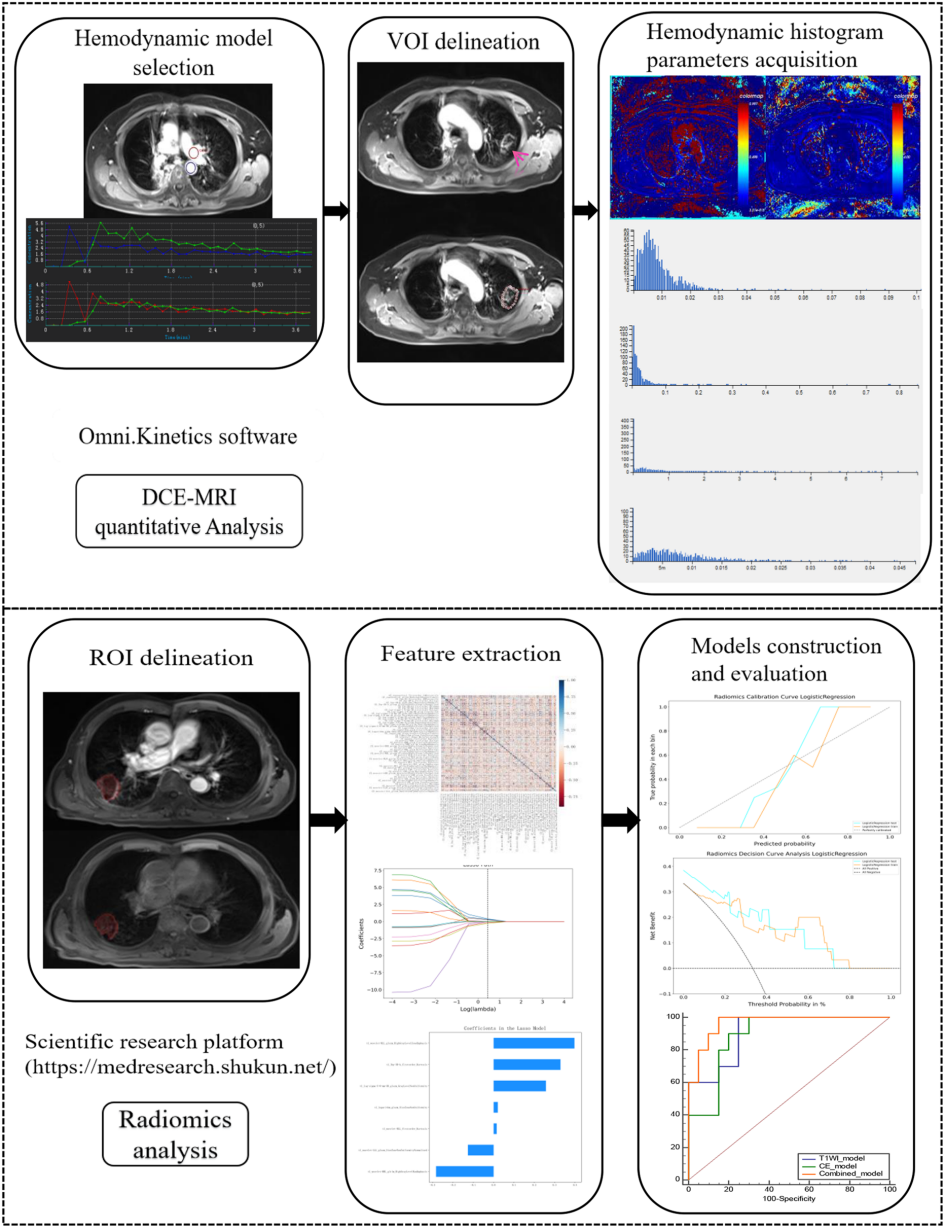
The study design

From January 2021 to October 2023, patients with malignant lung tumors treated with MWA in our hospital were enrolled (Fig. 1). The exclusion criteria were as follows: (1) Patients receiving other local treatments, such as radiotherapy; (2) Patients who did not undergo DCE-MRI examination at 24 h post-MWA; (3) Patients with a follow-up of less than 6 months; (4) Patients with insufficient image quality or incomplete DCE-MRI images. All primary lung cancers were confirmed by pathology, and lung metastases were confirmed by pathology or imaging follow-up. The flow diagram of the study was shown in Fig. 2.

### Computed tomography-guided MWA and follow-up studies

The settings of the 16-slice spiral CT scanner (Min-Found, China) were configured as follows: 16 × 1.2 collimation, 120 kV, 160 mAs, 0.5 S/R rotation speed, and a slice thickness of 2 mm. The microwave needle (FuZhong Medical Hi-tech Co, Ltd, China) was operated at a power of 30 W to 50 W with an ablation time ranging from 5 to 30 min. The ablation area completely covered the tumor and extended to include 0.5–1.0 cm of the surrounding normal lung tissue. After removing the needle, postoperative complications such as pneumothorax and hemorrhage were monitored.

Follow-up CT scans were performed immediately after MWA treatment, then every three months for one year after MWA treatment, and annually thereafter. The chest CT scan performed three months after MWA served as the baseline [24]. An interventional radiologist with over five years of ablation experience initially assessed the efficacy of MWA based on CT images taken at least six months after MWA treatment. The results were subsequently reviewed by another interventional radiologist with more than 20 years of experience in ablation procedures. The modified response evaluation criteria in solid tumors (m-RECIST) criteria [25, 26] targeted lesions with arterial phase enhancement (viable tumors). Complete response (CR): Complete disappearance of enhanced areas in all target and non-target lesions; Partial response (PR): A sum decrease of ≥ 30% in the diameters of the enhanced areas of target lesions; Stable disease (SD): Reduction does not reach PR or increase does not reach PD; Progressive disease (PD): A sum increase of ≥ 20% in the diameters of the enhanced areas of target lesions, or the appearance of new lesions. In accordance with the m-RECIST criteria, patients were classified into two groups: the effective treatment (CR + PR + SD) and the ineffective treatment (PD).

### DCE-MRI acquisition and image analysis

All patients underwent DCE-MRI examination within 24 h after MWA. One study indicated that the ablation area showed no significant enhancement in any phase of dynamic enhancement within 24 h after MWA surgery [27], which is of great significance for early detection of local tumor residue. Therefore, 24 h after MWA surgery was selected as the examination node.

They breathed freely and were scanned in the supine position. On a 3.0T Verio MRI scanner (Siemens AG, Erlangen Germany) via a 12-unit thoracic phased-array body coil, DCE-MRI datasets were obtained by 3D VIBE T1-weighted (T1W) dynamic perfusion sequences. Before the dynamic enhancement scan, a multi-flip angle scan was performed (TR = 3.25 ms, TE = 1.17 ms, flip angles = 5°, 10°, 15°, field of view = 350 mm × 282 mm, matrix size = 162 × 288, slice thickness = 5 mm, number of slice = 30, and temporal resolution = 6.5 s per cycle). The parameters of the dynamic enhancement scan sequence for scanning 35 phases were as follows: flip angle = 10 degrees, and the rest of the parameters were consistent with the description above. The total time of above two scans was 247 s. The control agent was gadodiamide (Omniscan, GE Healthcare) at an injectable dose of 0.1 mmol/kg and an injection rate of 2.5–3.0 ml/s.

DCE-MRI datasets were handled through Omni. Kinetics software (GE Healthcare, China). The free-breathing 3D correction technique (rigid-free medical image registration algorithm) was applied to rectify for motion artifacts. The smoother time intensity curve (TIC) after contrast-enhanced injection indicates a good fit. Perfusion parameters for dual bronchial and pulmonary artery supply were calculated using extend toft model (ETM) and exchange model (ECM). The upper and lower 3–5 layers of the outlined maximal tumor layer were merged into a ROI for quantitative analysis. ETM yielded four PK parameters (K^trans^. K_ep_, V_e_, V_p_) and ECM yielded five PK parameters (K^trans^. K_ep_, V_e_, V_p_ and F_p_). Histogram analysis of above PK parameters included skewness, kurtosis, uniformity, energy, entropy, Quantile5 (Q5), Q10, Q25, Q50, Q75, Q90, and Q95. All lesions were sketched by doctor 1 and nine lesions were randomly sketched by doctor 2 to evaluate the inter-rater correlation coefficients (ICC). They were spatially and temporally independent, and neither was aware of the medical history and the follow-up results.

### Radiomics analysis

Scientific research platform (https://medresearch.shukun.net/) was used for radiomics analysis.

#### Images delineation

In this study, MR images, acquired within 24 h post-MWA for malignant lung tumors, were uploaded to the Scientific research platform in DICOM format. The focus was on T1W and Ce-T1W images, with a particular emphasis on the ablation zones as targets. Doctor 1 with five years of experience manually delineated ROI on each slice, meticulously avoiding major blood vessels and bronchi. Subsequently, the software automatically generated three-dimensional (3D) reconstructions of the lesion. All lesions were sketched by doctor 1 and a radiologist with 20 years of experience confirmed the segmentation results. Twenty lesions were randomly sketched by doctor 2 to evaluate the ICC. They were spatially and temporally independent, and blind to the medical history and the follow-up results.

#### Data extraction

A total of 1874 radiomics features in the ablation area were calculated from T1W and Ce-T1W images of each lesion, respectively. There are 14 shape based features. Seven categories of features were used for 12 image preprocessing techniques (exponential, gradient, lbp-2D, lbp-3D-k, lbp-3D-m1, lbp-3D-m2, log-sigma, logarithm, original, square, square root, wavelet), including 360 first order features, 480 Gy-level co-occurrence matrix (GLCM), 280 Gy-level dependence matrix (GLDM), 320 Gy-level run-length matrix (GLRLM), 320 Gy-level size zone matrix (GLSZM), and 100 neighbouring gray tone difference matrix (NGTDM).

#### Model construction

When dealing with class-unbalanced datasets, the 7:3 grouping ensures that the class distribution in the training and test/validation groups is similar to that in the original dataset, thus reducing bias. Therefore, all lesions were divided into a training group and a test group in a ratio of 7:3 [28]. The training data was utilized for features selection and radiomics model construction. Initially, the features underwent normalization, and features with a Pearson correlation coefficient absolute value of ≥ 0.90 and ICC < 0.75 were excluded. Subsequently, Select *K* Best (*p* > 0.05) was employed to identify the *K* features with the highest scores, and then the least absolute contraction and selection operator (LASSO) was used to obtain the final features for logistic regression model construction. LASSO adjusted the Greek letter lambda (λ) to assign zero regression coefficients to uncorrelated features, and the selection of the best λ value involves 5 cross-validations, aiming to obtain the optimal model with the highest area under the curve (AUC) value of the cross-validations set.

#### Model evaluation

The performance of various signatures was verified using the test dataset, generating receiver operating characteristic (ROC) curves to calculate the corresponding AUC. The Delong test was used to compare predictive performance differences between the models. Additionally, sensitivity, specificity, accuracy, F1, positive predictive value (PPV), and negative predictive value (NPV) were also calculated. The Youden index determined the optimal cut-off value maximizing the sum of sensitivity and specificity. Calibration curves were plotted to assess calibration accuracy, alongside the Hosmer-Lemeshow (HL) test. Decision curve analysis (DCA) was also used to assess the clinical utility of the predictive signatures.

#### Survival analysis

The progression of lesions in ablation zone was defined as the primary endpoint. This was characterized by either a sum increase of ≥ 20% in the diameters of the enhanced areas of target lesions, or the emergence of new lesions. To evaluate patient outcomes based on radiomics features, survival curves were generated by Kaplan-Meier survival analysis. Subjects were categorized into two groups according to the cut-off value derived from each radiomics model: those above and those below the specified threshold. The statistical significance between the survival rates of these two cohorts was assessed using the *p*-value calculated from the comparison of their survival curves.

### Statistical analysis

The clinical and DCE-MRI datasets analysis was performed via SPSS 25.0 software (Version 25.0, NY). Quantitative data following to normal distribution were presented by a *T*-test and expressed as *X ± S*, or tested by a Wilcoxon test and expressed as median (upper and lower quartile). Categorical variables were analyzed by the *χ*^2^ test or *F-*test as appropriate. Image segmentation, features analysis, radiomics model construction and evaluation of statistical processing used scientific research platform (https://medresearch.shukun.net/). The “rms” package of R software (version 4.4.0, US) was used to construct calibration curves and DCA, and the “psych” package was used to calculate the ICC. The progression-free survival (PFS) of different risk cohorts was analyzed by Kaplan-Meier survival analysis. The significance threshold was set at *p* < 0.05.

## Results

### Characteristics of the study population

A total of 43 patients were finally enrolled in the study, including 28 cases with effective treatment and 15 cases with ineffective treatment. In our study, there were 12 cases of mild pneumothorax and 6 cases of mild bleeding on CT examination immediately after MWA, while there were 9 cases of mild pneumothorax, 6 cases of mild bleeding in DCE-MRI within 2 days after MWA.

The clinical characteristics are shown in Table 1. There was a significant difference in tumor size on CT between the effective and ineffective treatment cohorts (B = 0.147, OR = 1.159(95%CI 1.019,1.317)).

**Table 1:** Comparisons of patient characteristics

Characteristics	Effective treatment cohort (*n* = 28)	Ineffective treatment cohort (*n* = 15)	*P* value
Age	65.57 ± 11.43	66.07 ± 13.77	0.900
BMI	23.59 ± 2.54	24.07 ± 3.71	0.658
Gender			0.055
Female	17 (81.0%)	4 (7.3%)	
Male	11 (50.0%)	11 (50.0%)	
Location			1.000
Upper middle lobes	11 (64.7%)	6 (35.3%)	
Lower lobe	17 (65.4%)	9 (34.6%)	
Tumor size on CT (mm)	9.60 (7.60,14.38)	16.20 (10.60,20.80)	0.003^*^
Maximum diameter of ablation zone on CT (mm)	27.65 (26.05,42.10)	36.41 ± 10.41	0.169
Maximum diameter of ablation zone on T2 (mm)	36.00 ± 10.16	39.53 ± 9.95	0.280
Maximum diameter of ablation zone on T1 (mm)	32.19 ± 9.48	37.71 ± 10.19	0.084
Maximum diameter of ablation zone on DCE (mm)	32.89 ± 8.16	37.25 ± 10.24	0.135
Tumor pathology			0.674
Primary	8 (57.1%)	6 (42.9%)	
Metastasis	20 (69.0%)	9 (31.0%)	
-Colorectal cancer	10	7	
-Liver cancer	2	2	
-Gastric cancer	1	0	
-Breast cancer	3	0	
-Lung cancer	2	0	
-Pancreatic cancer	1	0	
-Nasopharyngeal carcinoma	1	0	

^*^p < 0.05 (two-sided) was considered statistically significant

### Differences of Pharmacokinetic parameters and their histogram parameters between the effective and ineffective treatment groups

**Fig. 3:**
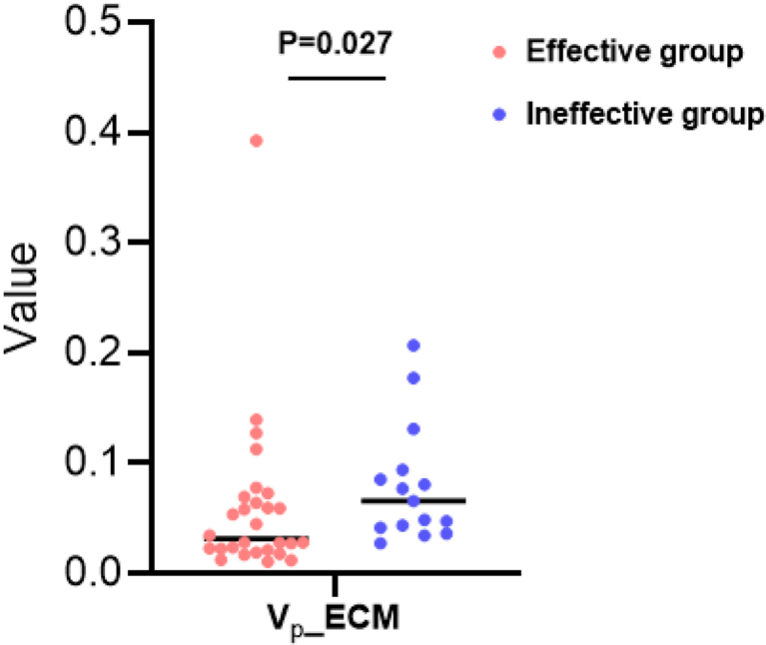
Scatter plot of the mean value of V_p_ from exchange model (ECM) between the effective group and the ineffective group. The mean value of V_p_ from ECM was 0.031 (0.022,0.068) and 0.065 (0.042,0.094), respectively

Four (K^trans^, K_ep_, V_e_, V_p_) and five (K^trans^, K_ep_, V_e_, V_p_, F_p_) PK parameters were obtained from the ETM and ECM, respectively. The mean value of V_p_ from ECM in the effective treatment group was statistically lower than that in the ineffective treatment group (Fig. 3). The ICC of V_p_ from ECM was 0.875.

A total of 108 histogram parameters were obtained from nine PK parameters by the two models. The ETM and ECM respectively obtained 11 and 10 histogram parameters with significant differences between the effective and ineffective treatment groups (shown in Table 2). The quantiles were higher in the ineffective group than in the effective group. Skewness and kurtosis of V_p_ from ETM and F_p_ from ECM in the ineffective group were higher than that in the effective group. Compared to the ineffective group, the effective group had higher energy and lower entropy of V_e_ (from ETM), but it had the lower energy and higher entropy of V_p_ (from ETM). The ICC of the above parameters ranged from 0.580 to 0.959, among which the ICC of six parameters (Q75 of K_ep_ from ETM, Q25, Q50, Q90, and Q95 of V_p_, and kurtosis of F_p_ from ECM) ranged from 0.500 to 0.750, and the ICC of two parameters (Q95 of K^trans^ and F_p_ from ECM) were higher than 0.900.

**Table 2:** The difference between effective and ineffective group of PK histogram parameters

Models	PK parameters	Histogram parameters	Effective treatment cohort (*n* = 28)	Ineffective treatment cohort (*n* = 15)	*P* value	ICC
ETM	K^trans^	Quantile75	0.032 (0.019,0.050)	0.059 (0.024,0.128)	0.047	0.789
		Quantile90	0.046 (0.028,0.082)	0.103 (0.038,0.202)	0.047	0.813
		Quantile95	0.058 (0.032,0.109)	0.134 (0.501,0.271)	0.039	0.837
	K_ep_	Quantile75	0.887 (0.607,1.446)	1.587 ± 0.809	0.044	0.602
	V_e_	Energy	0.059 (0.036,0.104)	0.038 (0.023,0.052)	0.044	0.836
		Entropy	4.553 ± 0.897	5.204 ± 0.835	0.025	0.898
	V_p_	Skewness	1.645 ± 0.949	2.874 ± 1.740	0.020	0.835
		Kurtosis	3.348 (0.297,5.938)	6.737 (2.168,15.044)	0.013	0.804
		Uniformity	-0.411 (-0.604,0.022)	-0.919 ± 0.715	0.013	0.798
		Energy	0.174(0.071,0.256)	0.338 ± 0.204	0.025	0.821
		Entropy	4.876 ± 1.214	3.886 ± 1.545	0.026	0.849
ECM	K^trans^	Quantile95	0.148 (0.086,0.209)	0.206 (0.134,0.697)	0.037	0.959
	K_ep_	Quantile95	3.252 ± 1.581	4.222 ± 1.303	0.049	0.820
	V_p_	Quantile25	0.004 (0.002,0.010)	0.008 (0.003,0.018)	0.037	0.708
		Quantile50	0.011 (0.005,0.022)	0.029 ± 0.023	0.018	0.580
		Quantile75	0.022 (0.015,0.048)	0.042 (0.021,0.063)	0.030	0.772
		Quantile90	0.034 (0.023,0.076)	0.066 (0.038,0.098)	0.022	0.700
		Quantile95	0.043 (0.029,0.095)	0.086 (0.047,0.123)	0.018	0.658
	F_p_	Skewness	1.052 ± 0.635	1.514 (1.070,2.126)	0.027	0.841
		Kurtosis	1.087 (-0.010,2.315)	2.455 (0.954,5.100)	0.030	0.602
		Quantile95	0.120 (0.071,0.158)	0.189 (0.106,0.349)	0.039	0.959

Note: PK refers to pharmacokinetic; ICC refers to inter-rater correlation coefficients

### Diagnostic efficacy of Pharmacokinetic parameters of DCE-MRI

**Fig. 4:**
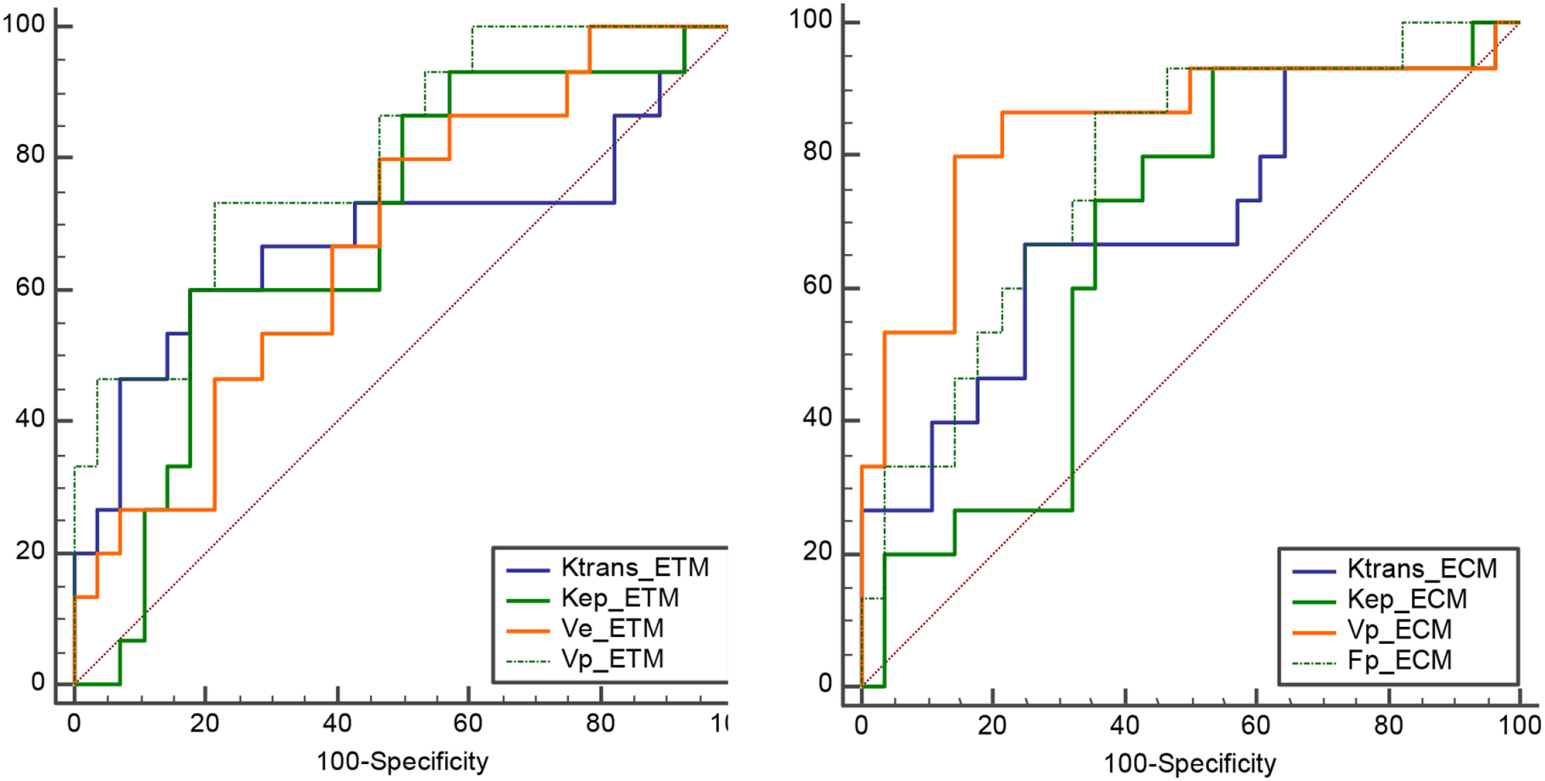
Receiver operating characteristic (ROC) curves of above eight pharmacokinetic (PK) histogram parameters. The AUCs of K^trans^, K_ep_, V_e_, and V_p_ from extend toft model (ETM) were 0.683, 0.688,0.676, and 0.805, respectively. The AUCs of K^trans^, K_ep_, V_p_ and F_p_ from exchange model (ECM) were 0.695, 0.667, 0.843, and 0.776, respectively

**Fig. 5:**
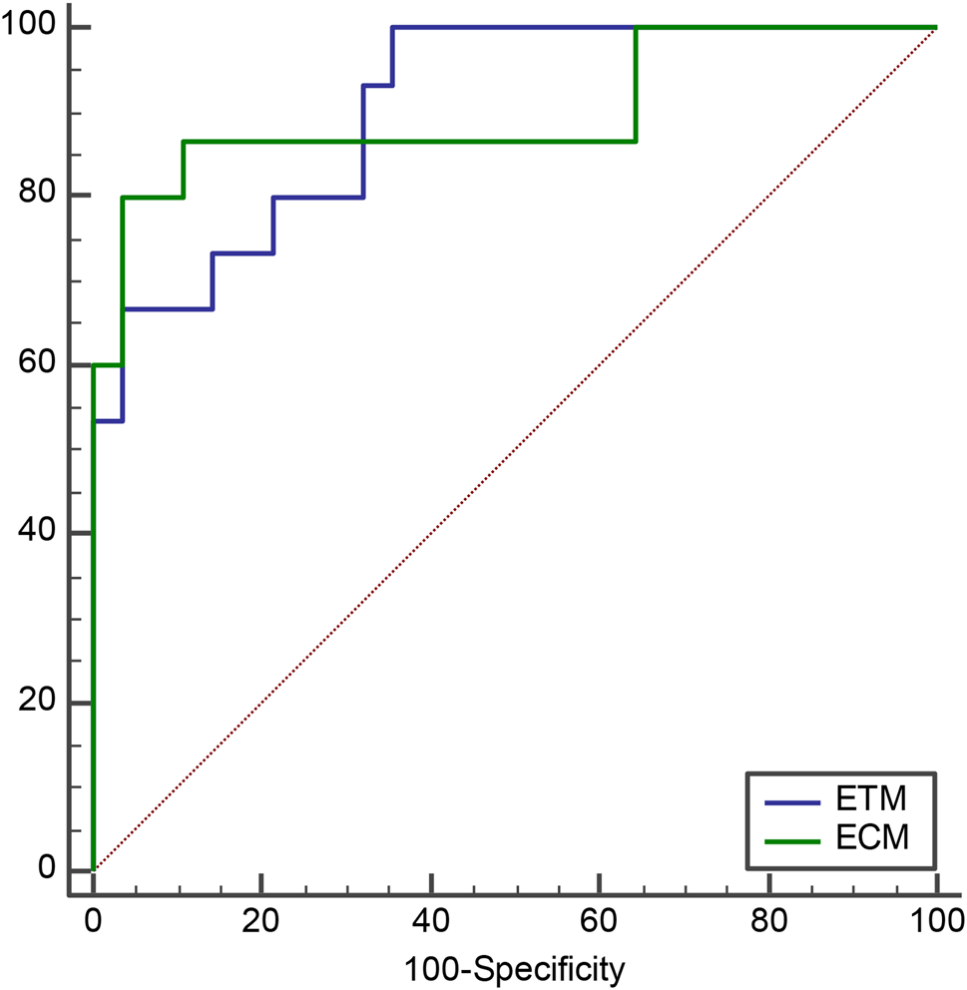
Receiver operating characteristic (ROC) curves of extend toft model (ETM) and exchange model (ECM). The AUCs of ETM and ECM was 0.905 and 0.900, respectively

The ROC curves with AUC, sensitivity, specificity and Youden index of above PK histogram parameters were shown in Fig. 4; Table 3. We found that the AUCs of V_p_ histogram parameters were higher than other parameters (AUC = 0.805 for ETM, and AUC = 0.843 for ECM). The AUCs of ETM and ECM was 0.905 (95%CI 0.776, 0.973) and 0.900 (95%CI 0.770, 0.970), respectively (shown in Fig. 5). The sensitivity, specificity and Youden index were 1.000, 0.643, and 0.643 for ETM and 0.800, 0.964, and 0.764 for ECM, respectively.

**Table 3:** Diagnostic efficacy of above eight PK histogram parameters

PK histogram parameters	AUC	Sensitivity	Specificity	Youden index
K^trans^_ETM	0.683 (95%CI 0.524,0.817)	0.600	0.821	0.421
K_ep__ETM	0.688 (95%CI 0.529,0.820)	0.600	0.821	0.421
V_e__ETM	0.676 (95%CI 0.516,0.811)	0.800	0.536	0.336
V_p__ETM	0.805 (95%CI 0.655,0.910)	0.733	0.786	0.519
K^trans^_ECM	0.695 (95%CI 0.536,0.826)	0.667	0.750	0.417
K_ep__ECM	0.667 (95%CI 0.507,0.803)	0.933	0.464	0.398
V_p__ECM	0.843 (95%CI 0.700,0.936)	0.800	0.857	0.657
F_p__ECM	0.776 (95%CI 0.623,0.889)	0.867	0.643	0.510

Note: K^trans^_ETM, K_ep__ETM, V_e__ETM, and V_p__ETM refered to K^trans^ (Q75, Q90,Q95), K_ep_ (Q75), V_e_ (energy, entropy) and V_p_ (skewness, kurtosis, uniformity, energy, entropy) from ETM. K^trans^_ECM, K_ep__ECM, V_p__ECM, and F_p__ECM refered to K^trans^ (Q95), K_ep_ (Q95), V_p_ (Q25, Q50, Q75, Q90, Q95), F_p_ (skewness, kurtosis, Q95) from ECM, respectively. AUC = area under curve

### Radiomics characteristics

The training cohort included 30 patients (20 effective treatment vs. 10 ineffective treatment), and the test cohort included 13 patients (8 effective treatment vs. 5 ineffective treatment). Seven features from T1WI and five features from Ce-T1WI were finally selected. When we combined T1WI with Ce-T1WI features, 10 features were eventually enrolled, including four features for Ce-T1WI and six features for T1WI (Table 4).

**Table 4:** The features selected from T1WI, Ce-T1WI and combined sequences

Sequences	Coefficients	Image preprocessing	Categories	Features
T1WI	0.397	wavelet-HLL	GLSZM	High Gray Level Zone Emphasis
	0.329	lbp-3D-k	Firstorder	Kurtosis
	0.257	log-sigma-3-0-mm-3D	GLSZM	Gray Level NonUniformity
	0.020	logarithm	GLSZM	Size Zone NonUniformity
	0.015	wavelet-HLL	Firstorder	Kurtosis
	-0.127	wavelet-LLL	GLSZM	Size Zone NonUniformity Normalized
	-0.283	wavelet-HHL	GLRLM	High Gray Level Run Emphasis
Ce-T1WI	1.201	wavelet-LLH	Firstorder	Skewness
	0.797	gradient	Firstorder	10 Percentile
	0.622	logarithm	GLSZM	Gray Level NonUniformity
	0.370	wavelet-LLH	GLCM	Cluster Prominence
	-0.234	wavelet-LLH	Firstorder	10 Percentile
Combined	1.589	wavelet-LLH	Firstorder	Skewness_Ce-T1
	1.216	wavelet-HLL	GLSZM	High Gray Level Zone Emphasis_T1
	0.821	lbp-3D-k	Firstorder	Kurtosis_T1
	0.644	gradient	Firstorder	10 Percentile_Ce-T1
	0.592	logarithm	GLSZM	Gray Level NonUniformity_Ce-T1
	0.265	log-sigma-3-0-mm-3D	GLSZM	Gray Level NonUniformity_T1
	0.154	wavelet-HLL	Firstorder	Kurtosis_T1
	-0.109	logarithm	GLSZM	Size Zone NonUniformity_T1
	-1.245	wavelet-LLH	Firstorder	10 Percentile_Ce-T1
	-1.939	wavelet-HHL	GLRLM	High Gray Level Run Emphasis_T1

GLSZM refers to gray-level size zone matrix, GLRLM refers to gray-level run-length matrix, and GLCM refers to gray-level co-occurrence matrix

### Performance of the radiomics models based on MRI

**Fig. 6:**
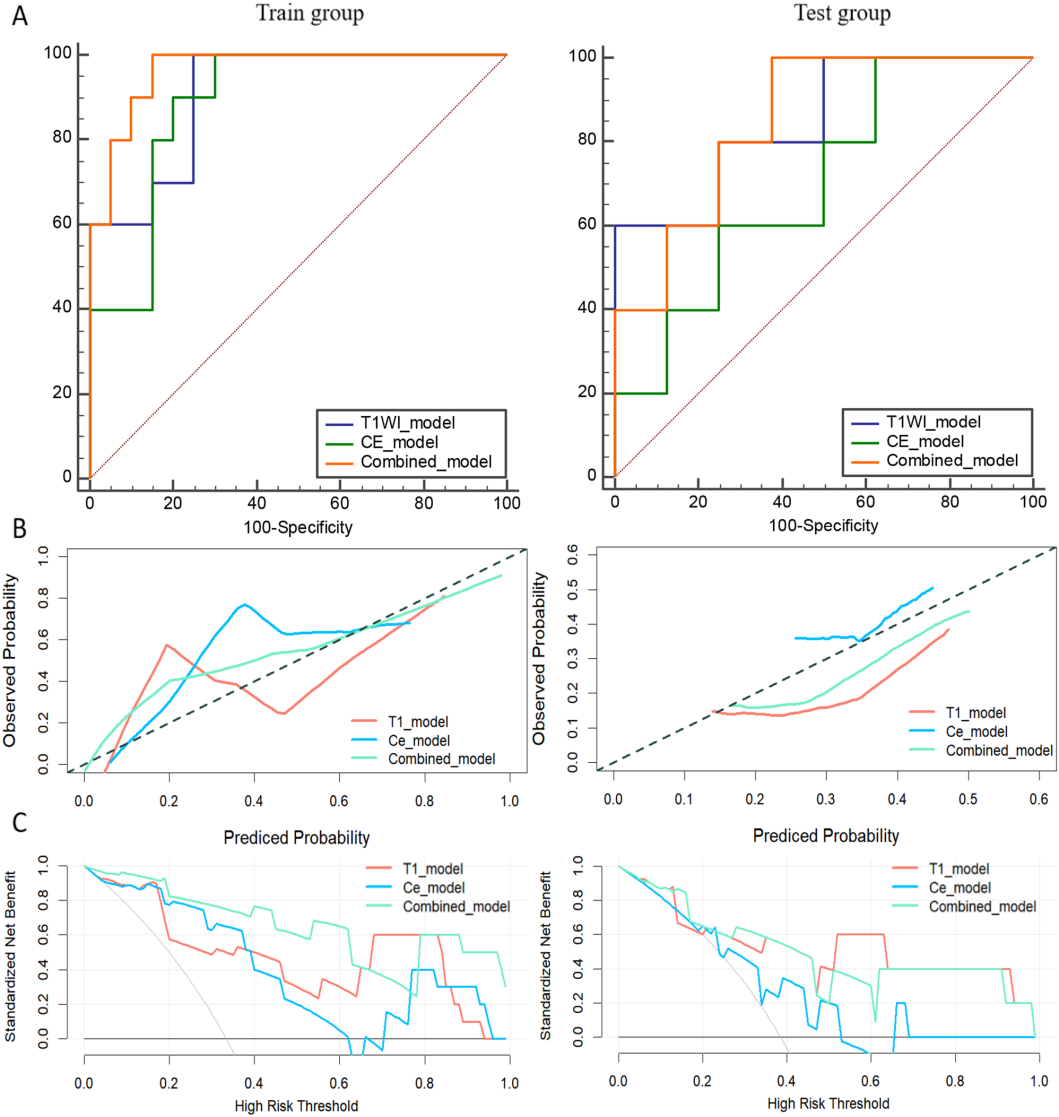
The Receiver operating characteristic (ROC) curves (**A**), calibration curves (**B**), and decision curve analysis (**C**) of T1WI, Ce-T1WI (CE) and combined models in the train and test groups

Three radiomics models based on T1WI, Ce-T1WI and combined sequences were constructed. The diagnostic performance of three models in the training and test groups were listed in Table 5. When three MRI-based radiomics models had the same sensitivity, the combined model had the highest specificity and accuracy in the training and test groups. The ROC curves, calibration curves, and DCA of three models were shown in Fig. 6.

**Table 5:** The diagnostic performances of three models in the training and test groups

	Training group			Test group		
	T1WI	Ce-T1WI	Combined	T1WI	Ce-T1WI	Combined
AUC	0.910(95%CI 0.748,0.983)	0.890(95%CI 0.722,0.974)	0.965(95%CI 0.825,0.999)	0.850(95%CI 0.550,0.982)	0.700(95%CI 0.393,0.913)	0.850(95%CI 0.550,0.982)
Sensitivity	1.000	1.000	1.000	0.800	0.800	0.800
specificity	0.750	0.700	0.850	0.625	0.500	0.750
Accuracy	0.833	0.800	0.900	0.692	0.615	0.769
F1	0.800	0.769	0.870	0.667	0.615	0.727
Youden index	0.750	0.700	0.850	0.600	0.375	0.625
PPV	0.667	0.625	0.769	0.571	0.500	0.667
NPV	1.000	1.000	1.000	0.833	0.800	0.857

Note: PPV refers to Positive predictive value; NPV refers to Negative predictive value

### The role of radiomics features in predicting survival outcomes

**Fig. 7:**
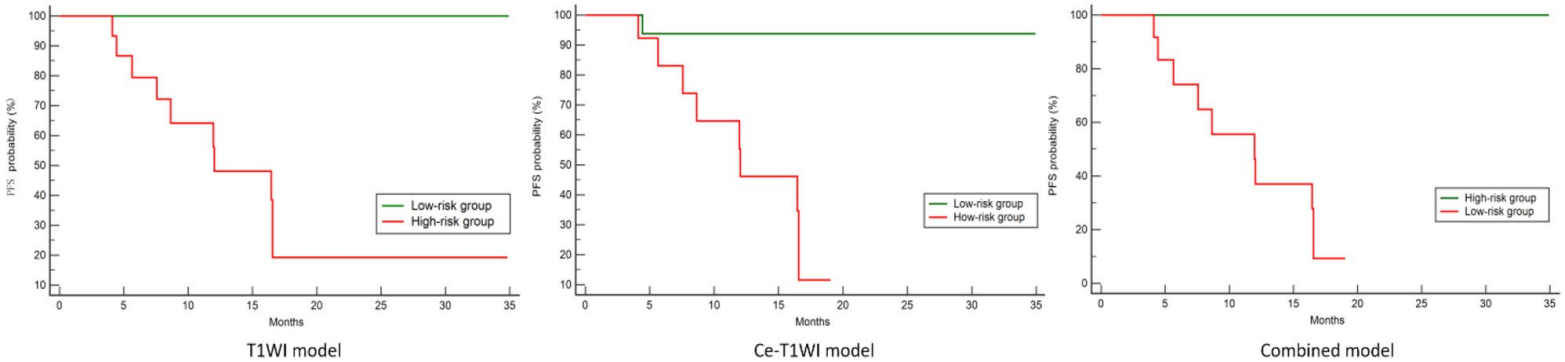
In the training cohort, survival curves were plotted based on patients whose Nomo-scores were above or below the cut-off values of the T1WI model (*P* < 0.001), Ce-T1WI model (*P* < 0.001), and combined model (*P* < 0.001). The red line represented the high-risk group, while the green line signified the low-risk group

PFS for the effective and ineffective groups was 477.0 (244.3, 770.0) and 344.0 *±* 189.6 days, respectively (*p* = 0.108). For the T1WI, Ce-T1WI, and combined models, the optimal cut-off points, based on the sum of sensitivity and specificity, were determined to be Nomo-scores of 0.256, 0.273, and 0.455, respectively. Patients were stratified into high-risk (Nomo-score below the cut-off) and low-risk (Nomo-score above the cut-off) groups using the corresponding optimal cut-off values. In the training cohort, the PFS of the low-risk group was significantly longer than that of the high-risk group (Fig. 7).

### Discussion

The aim of this study was to investigate the application of quantitative DCE-MRI analysis and MRI-based radiomics models in assessing the efficacy of LC patients after MWA. Quantitative parameters derived from the analysis of tracer kinetic models (ETM and ECM), which correlate with tissue perfusion and capillary permeability, provide valuable insights into tumor status after local MWA treatment. During a follow-up period of at least six months, we found differences in PK parameters and their histogram parameters in LC patients with different prognoses. In addition, we developed three radiomics models: one based on T1WI, one based on Ce-T1WI, and a combined model combining the former two to predict the efficacy of LC patients after MWA. The results of the study showed that the MRI radiomics model exhibited high specificity and accuracy.

Quantitative PK parameters have been used to assess tumor response to interventional therapy [29–32]. After MWA, tumors undergo coagulative necrosis, resulting in reduced tissue perfusion and decreased capillary permeability, which should be reflected in lower perfusion parameter values. V_p_ denotes the volume fraction of plasma per unit volume, and it has been suggested that V_p_ might be useful in the early prediction of disease progression [33]. In this study, the V_p_ value was significantly higher in the ineffective group compared to the effective group, suggesting that higher V_p_ value from ECM may be associated with poorer treatment effect. Histogram parameters provided complementary data to measure the response to tumor treatment [34–36]. Skewness, kurtosis, homogeneity, entropy, energy, and quantiles are commonly used. Quantiles refer to the configuration of the histogram, while skewness and kurtosis describe the degree and tendency of the grayscale distribution to deviate from symmetry, respectively. Changes in histogram contours and asymmetry reveal differences in the tumor microenvironment. Previous studies have shown that skewness, kurtosis, and entropy correlate with tumor heterogeneity [37–39]. Our results showed that the skewness and kurtosis of V_p_ from ETM and F_p_ from ECM in the ineffective group exceeded those in the effective group, whereas the quantiles of other PK parameters (especially higher quantiles such as Q95 and Q75) were higher in the ineffective group, except for V_p_ from ETM. These statistical measures suggested that the distribution of these parameters were more variable and potentially more higher in the ineffective group. In the histogram parameters of V_e_ from ETM, the ineffective group had higher energy and lower entropy than the effective group; in the parameters of V_p_ from ETM, the ineffective group had lower energy and higher entropy than the effective group. Energy and entropy are textural features that can reflect the uniformity and complexity of the MR signal within the ROI, respectively. Together, these results suggested that the tissue perfusion and the tumor heterogeneity in the ineffective group were higher than those in the effective group after MWA, which might be related to the incomplete ablation of tumors and the high invasiveness of the tumors in the ineffective group. We also observed that the histogram parameters of V_p_ appeared to be superior to other parameters in predicting the efficacy of MWA, with AUCs of 0.805 and 0.843, respectively. Therefore, the PK parameters and their histogram attributes of DCE-MRI may predict tumor efficacy after MWA to a certain extent, especially V_p_ and its related histogram parameters.

Among the MR multiple sequences, we first selected the pressurized lipid T1W, T2W, and Ce-T1 sequences to outline the ROI of the lesion. During the outlining process, we found that the signals of hemorrhage and inflammatory edema in T2WI were more difficult to differentiate, and the stability of model efficacy in the training and test groups was poorer (see Additional file 1), so we did not consider the T2WI model in the later study. In this experiment, we extracted seven and five radiomics features from T1WI and Ce-T1WI, respectively. The features from T1WI were mainly features of GLSZM, firstorder, and GLRLM via wavelet, lbp, and log transformations. Among them, kurtosis, gray level non uniformity, size zone non uniformity, and high gray level run emphasis were reproduced in a study of CT imaging histological model to assess the efficacy of lung malignancy after MWA [18, 40, 41]. Kurtosis reflects the degree of grayness. Other parameters measure textural and spatial heterogeneity. Features from Ce-T1WI were mainly firstorder, GLSZM, and GLCM features derived from wavelet, log, and gradient transformations, where 10 percentile, gray level non uniformity, and cluster prominence have also appeared in previous studies [40, 41]. The above parameters mainly reflect the degree of gray in-homogeneity, and the feature selection process helps in identifying the most informative parameters for distinguishing between the effective and ineffective treatments. Our recent study concluded that CT imaging histology for predicting the efficacy of MWA in lung malignancies was feasible and reliable by performing imaging histology analysis on preoperative [40], ΔCT [41], and postoperative [42] images of MWA. Huang et al. combined preoperative and postoperative CT images and reached a similar conclusion by habitat analysis [43]. As most patients with MWA of lung tumors are treated with the aim of cure, CT follow-up shows a significant radiological burden. Replacement of one or more CT with MRI can significantly reduce the cumulative dose in these patients. In addition, in our previous study, some tumors were adjacent to blood vessels, and pulmonary hemorrhage was inevitable during MWA, and when outlining the ROI, it was more difficult to distinguish the ablation area ground glass shadow from the hemorrhagic foci on the CT image. MRI compensated for this shortcoming well, and in predicting the efficacy, the T1WI model also showed a better efficacy than the CT model. Six of the image features constituting the combined model were from T1WI and four were from Ce-T1 sequences. According to the coefficient weights of each feature, it was known that the T1WI features might have a greater influence on the model. The AUC values for the T1WI model, Ce-T1WI model, and combined model were calculated for both the training and test groups. In the training group, the AUCs were 0.910, 0.890, and 0.965, respectively, indicating high discriminative power for all models, especially the combined model. In the test group, the AUCs were 0.850, 0.700, and 0.850, respectively, showing a slight decrease in performance but still maintaining reasonable accuracy, particularly for the T1WI and combined models. These results highlighted the potential of using advanced MRI analysis technique to evaluate the efficacy of MWA in LCs. The combination of different MRI sequences and derived features improved the predictive power of the model.

Survival curves were utilized to visually assess early outcomes after MWA in patients with lung tumors, stratified into high-risk and low-risk groups. In the training cohort, significant differences were observed in survival curves between patients above and below the Nomo-scores threshold for the T1WI, Ce-T1WI, and combined models. These findings highlighted the potential utility of radiomic features in survival modeling. However, due to the relatively small sample size in the current study, no significant difference in the survival curve was observed between high-risk and low-risk groups in the test cohort.

Conventional imaging features were not included in this study because MRI is less useful than CT for showing peri-tumor structures. Previous studies have shown that tumor size was an independent variable in the early efficacy of MWA for the treatment of lung malignancies, and patients with large tumors had a lower survival rate after MWA than patients with small tumors [44, 45]. In the present study, the mean tumor size was 9.6 mm and 16.2 mm in the effective and ineffective groups, respectively, which is consistent with previous reports. In clinical practice, there are indeed differences in the prognosis of lung cancers originating from various sources. However, our study observed no significant statistical difference in the prognosis between patients with primary lung cancer and pulmonary metastases after MWA.

The reprehensibility and stability of image parameters for manual and semi-automatic sketching have always been a concern. In this study, the ICC of DCE-MRI perfusion parameters ranged from 0.580 to 0.959, and most of the parameters showed high repeatability (ICC ≥ 0.75), and six parameters (Q75 of K_ep_ from ETM, Q25, Q50, Q90 and Q95 of V_p_ from ECM, and kurtosis of F_p_ from ECM) showed moderate repeatability (0.75 > ICC ≥ 0.5) [46]. Radiomics features (ICC < 0.75) were excluded, and all features used for feature screening and model building had great repeatability.

There are several limitations of this study: Firstly, due to ethical requirements and timeliness, we did not perform pre- and post-treatment DCE-MRI in the treatment group. Secondly, the overall sample size was small and patients in this study came from the same hospital, which was prone to bias. More cases which come from different hospitals will be included for further study in the future. Thirdly, although there was no difference in prognosis between primary lung cancer and lung metastases in this study, for the sake of the rigor of the study, we will refine lung cancer from different sources and conduct separate studies for validation in the future. In the end, the accuracy of quantitative parameters is affected not only by the choice of hemodynamic model, but also by the type of contrast agent, injection protocol, scan time resolution, scan time, and image noise level.

Overall, this study highlighted the potential of quantitative DCE-MRI analysis and MRI-based radiomics models in evaluating the efficacy of MWA in treating patients with LCs. This study laid the foundation for further exploration of the application of DCE-MRI and radiomics in assessing and predicting the efficacy of various oncological interventions in the future.

## Electronic supplementary material

Below is the link to the electronic supplementary material.

Supplementary Material 1

## Data Availability

The datasets used and/or analysed during the current study are available from the corresponding author on reasonable request.

## Abbreviations

AUC: Area under the curve
Ce-T1: Contrast-Enhanced T1
CR: Complete response
DCA: Decision curve analysis
DCE-MRI: Dynamic Contrast-Enhanced MRI
ECM: Exchange model
ETM: Extend toft model
GGO: Ground glass opacity
GLCM: Gray-level co-occurrence matrix
GLDM: Gray-level dependence matrix
GLRLM: Gray-level run-length matrix
GLSZM: Gray-level size zone matrix
HL: Hosmer-Lemeshow
ICC: Inter-rater correlation coefficients
LASSO: Least absolute contraction and selection operator
LC: Lung cancer
MWA: Microwave Ablation
NGTDM: Neighbouring gray tone difference matrix
NPV: Negative predictive value
NSCLC: Non-small cell lung cancer
PD: Progressive disease
PFS: Progression-free survival
PK: Pharmacokinetic
PPV: Positive predictive value
PR: Partial response
Q5: Quantile5
ROC: Receiver operating characteristic
SD: Stable disease
T1W: T1-weighted
TIC: Time intensity curve
λ: The Greek letter lambda
