# Supplementary material for: DCE-MRI quantitative analysis and MRI-based radiomics for predicting the early efficacy of microwave ablation in lung cancers
Source: Cancer Imaging. 2025 Mar 10;25:26. doi: 10.1186/s40644-025-00851-7 (PMC11892232; doi:10.1186/s40644-025-00851-7)
Supplement: Supplementary file 1 — Supplementary Material 1 [file 40644_2025_851_MOESM1_ESM.doc]

**Additional file 1**


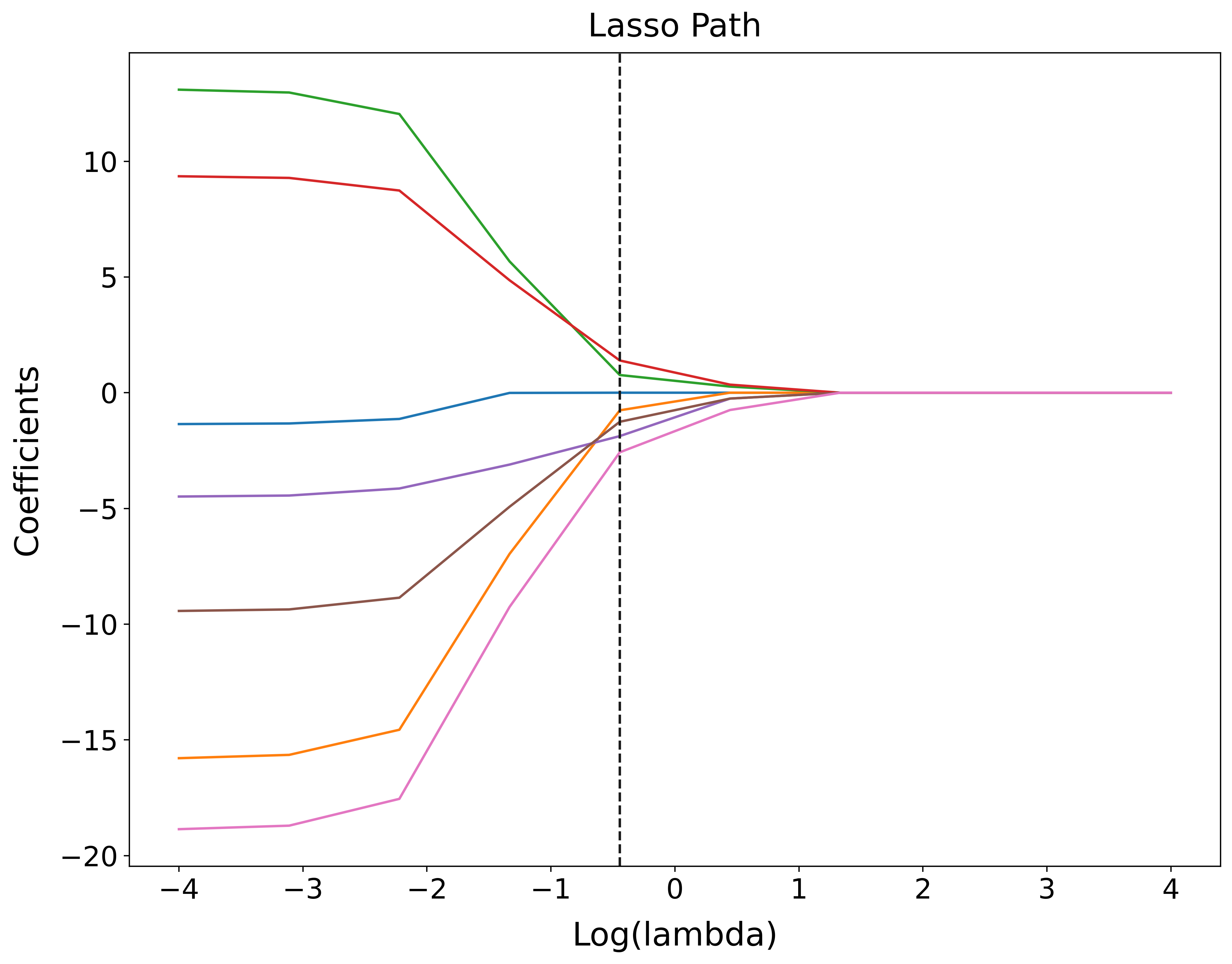


**Fig.1 The Lasso path**

**Table 1 The features selected fromT2WI**

| Sequences | Coefficients | Image preprocessing | Categories | Features |
| --- | --- | --- | --- | --- |
| T2WI | 1.392 | logarithm | glszm | Gray Level Non Uniformity Normalized |
|  | 0.764 | log-sigma-3-0-mm-3D | glszm | Small Area High Gray Level Emphasis |
|  | -0.761 | log-sigma-3-0-mm-3D | glszm | Size Zone Non Uniformity |
|  | -1.256 | wavelet-LHL | firstorder | Minimum |
|  | -1.875 | wavelet-HHH | glrlm | Gray Level Non Uniformity Normalized |
|  | -2.575 | wavelet-LHL | firstorder | Skewness |

**Table 2 The diagnostic performances of T2WI models in the training and test groups**

|  | Training group | Test group |
| --- | --- | --- |
| AUC | 0.985(95%CI 0.748,0.983) | 0.375(95%CI 0.550,0.982) |
| Sensitivity | 1.000 | 0.600 |
| specificity | 0.900 | 0.250 |
| Accuracy | 0.933 | 0.385 |
| F1 | 0.909 | 0.429 |
| PPV | 0.833 | 0.333 |
| NPV | 1.000 | 0.500 |
